# Exploring information needs among family caregivers of children with intellectual disability in a rural area of South Africa: a qualitative study

**DOI:** 10.1186/s12889-024-18606-7

**Published:** 2024-04-24

**Authors:** Mantji Juliah Modula, Mpho Grace Chipu

**Affiliations:** School of Nursing, Faculty of Health Sciences, University of Free State, Nelson Mandela Drive, 9301 Bloemfontein, Free State Province South Africa

**Keywords:** Caregivers, Children, Family, Information needs, Intellectual disability, Rural, South Africa

## Abstract

**Background:**

Globally, families experience challenges caring for and raising children with intellectual disability (ID). Family caregivers in rural states are mostly known for lacking support resources, including information on understanding the care of ID. Lack of adequate information on understanding of ID compromises the provision of life-long care and support of the children with ID’s physical, emotional, psychological and social developmental well-being. The study aimed to explore the information needs of family caregivers regarding the care of children with ID in rural areas of Limpopo Province, South Africa.

**Methods:**

This qualitative explorative research conducted 16 in-depth individual interviews and one focus group discussion with ten family members. The participants shared their experiences of raising children with ID in rural communities. Inductive thematic analysis using Atlas Ti software categorised emerging themes and subthemes of this study from merged data sets on information needs regarding the care of children with ID among family caregivers.

**Results:**

The findings highlighted the need for information regarding ID care among family caregivers raising children with ID in the home environment. The information challenges experienced by family caregivers include caring for the challenging behaviour of children with ID and available support resources and services for the children and their families. These challenges impact the care and support required to meet the developmental needs of children with ID. Furthermore, inadequate information on ID among family caregivers in rural communities with a lack of resources restricts the children from accessing required support services.

**Conclusions:**

Given the information challenges these families face on ID, the stakeholders must develop continuous training programmes that will equip, empower, and further monitor ID care and management among family caregivers to enhance care and the raising of children with dignity.

**Supplementary Information:**

The online version contains supplementary material available at 10.1186/s12889-024-18606-7.

## Background

Intellectual disability (ID) diagnosis requires families as primary caregivers to support their children during the developmental stages of life. ID as a neurodevelopmental disorder limits intellectual functioning and adaptive behaviour during the developmental period of a child [1]. ID ranges from mild, moderate, severe and profound impairment. The diagnosis affects the child’s communication, cognitive and self-care skills development, leading to dependency support living [2]. Children with ID are prone to physical conditions, including obesity and constipation, often exposing them to misdiagnosis and underdiagnosis [3], limiting their access to healthcare services. Early diagnosis of ID enables early intervention and enhances acceptance of the child by the community [4].

Globally, ID affects approximately 2–3% of the general population [5], and the prevalence of children under five is 3.2% [6]. The incidence in low-income countries is 16.41/1000; in middle-income countries, it is 15.94/1000 persons, higher than in other countries, with approximately 9.21/1000 persons [4]. South Africa experiences a lack of data on the prevalence of ID and is further dependent on the census.

ID diagnosis generally increases the lifelong dependency of the children on their families regarding self-care and support to thrive. As a primary caregiver, every family is an environment where essential interpersonal experiences of a child’s development occur [7], including personality, behaviour, and attitudes. Even though families take the lead in raising these children, ID awareness and information among the carers has been a continued concern [8]. In addition, information created for childrearing may not benefit caregivers of children with ID [9]. In most cases, families of children with ID experience parenteral barriers, including limited knowledge related to a lack of resources or support [10].

Past research established that children with ID are more at risk of experiencing childhood problems than their peers without ID [11]. As most children with ID have a prevalence of developmental delay impacting their independence ability [12], their care requires information and understanding of ID. Past research has shown that adequate knowledge is measured through the caregiver’s confidence in a particular realm or topic to declare the knowing of what, how, when and why questions that inform one’s attitudes and further influence one’s behavioural changes [13].

Quality caregiving of a child with ID influences their developmental outcomes [14]. It is noteworthy that parents’ understanding of the capacity to care for children with ID is assessed based on their responses to the needs of such children [15]. Previous studies highlighted how the families were more reactive than proactive regarding caring for their children with ID, leading to social exclusion by their communities [16]. Although the need for training of parents raising children with ID has broadly been investigated successfully [17], exploring information needs on the care of ID among primary caregivers in rural areas has yet to be fully established.

South Africa surpassed an estimated population of 60 million, with more than 19 million people residing in rural jurisdictions. The country still needs improvement in service delivery problems, especially in rural states. The pastoral population’s life challenges are unique and mainly complex due to geographical and race/ethnicity characteristics [18]. Previous studies on the experiences of families of children with ID in the South African rural context revealed challenges related to poverty, poor education, and unemployment, including limited transport to access professional services and support systems for ID [19–22]. Furthermore, rural communities report low access to health services, including a scarcity of professionals and specialist care, exposing them to health risks resulting in shorter life expectancy [23, 24].

Evidence from previous research established that the diagnosis of children with ID in rural areas is likely delayed due to geographic barriers to access services, causing health disparity for children compared to their peers in urban areas [25]. This delay prevents families from receiving information relevant to planning and managing the child’s care, which is considered a way of simplifying the processes of the professionals [26]. The family caregivers of children with ID require information including health specialists and rehabilitation services for their children, government support grants, networking and educational services [27, 28]. The social model posits that the lack of information access experienced by caregivers of children with ID limits their parenting abilities and further impacts the support of the functioning level of their children [29]. The study aimed to explore the family caregivers’ information needs regarding understanding the care of children with ID in rural areas to fill the gaps in the caring and management challenges of ID in the home environment. Adequate information by families regarding the care of ID is vital in meeting the support needs of their children with ID in rural areas. These require comprehensive care, support, and rehabilitation for children and their families. The study’s objective was to explore and describe the information needs of family caregivers regarding the care of children with ID in rural areas of Limpopo Province, South Africa.

## Methods

### Study design

This qualitative explorative research helped the researcher facilitate data collection, analysis, and interpretation of the finding processes [30]. The approach enabled an unrestrained process that explored the participants’ information needs regarding the care of children with ID in a rural home environment. The focus group discussion and in-depth individual interviews allowed participants to share their experiences caring for and managing a child with ID in a rural community. The first researcher recruited participants, collected data, analysed, and wrote manuscripts. The second author reviewed the manuscript.

### Setting

The researcher conducted the study in the Capricorn district municipality of Limpopo Province, which is approximately 80% rural and estimated at 5.8. million population [31]. The district is one of the five municipalities of the province and has a growing economic centre. It is known for providing better community services such as job opportunities, schooling, and health care facilities in the province. Thus, it attracts a high population density with a unique cultural heritage, diverse ethnicities, and five languages under 30 traditional authorities [31, 32]. The district’s households in the rural community experience challenges regarding limited access to basic needs, including piped water, poor hygienic sanitation conditions and restricted access to health and education services. The high unemployment in this rural status contributed towards people migrating to other provinces for job opportunities, leaving most families to female and single-headed households [33].

### Participants and sampling

The study sampled 26 families from different tribal authorities of Capricorn district with the core function of directly caring for and raising children with ID in home environments. The vulnerability of family caregivers resulted in some hiding their children from their communities due to social stigma related to ID diagnosis. The researcher recruited participants whose children were accessing education and health facilities in their communities through purposive and snowballing sampling strategies. The strategies assisted the researcher in reaching those families not known to community facilities, including schools and health care services, as their children were not utilising such services. The family caregivers recruited, invited, and introduced other families who were relatives or neighbours or encountered them at churches or community meetings and met inclusion criteria to the researcher [30]. Such families’ children were diagnosed with ID from hospitals in other provinces and private health care services. They did not use public services, fearing discrimination related to stigmatisation from their communities.

Eligibility criteria included any family caregiver above 18 years who was living and directly involved with raising children with ID and was voluntarily willing to share their experiences regarding caregiving informational needs in the rural home environment. Furthermore, the researcher’s judgement stayed instrumental in the selection of family caregivers whose children were of school-going age or over six years old, indicating that they were not recently diagnosed with ID. For most children at this age, healthcare professionals have already established their diagnosis of ID, as their developmental capabilities were able to be assessed with their peers at school. Furthermore, on this ground, the researcher’s opinion assumed that children were emotionally ready to express their feelings in their life circumstances. The researcher further believed such participants had adequate information to relate their experiences as primary family caregivers raising children with ID. The researcher excluded family caregivers of children with IDs less than 6 and above 19 years old and those family members who did not live with the children in a home environment.

The family caregivers were between 19 and 67 years old, and most left schooling at the secondary level. The sample comprised 16 mothers, one father, two grandparents (one grandfather and one grandmother), three aunts, two uncles and two guardians of the children with ID. Table 1 below indicates the participants’ demographic information and the profile of children with ID.

**Table 1:** The socio-demographic data of family caregivers and the profile of children with ID

Variable	Category	Number of Participants (*n* = 26)	Percentage (100)
Participants’ gender	Females	22	85%
	Males	4	15%
Participant’s age distribution in years	19–28 years	3	12%
	29–38 years	4	15%
	39–48 years	11	42%
	49–58 years	5	19%
	59–68 years	3	12%
Participants’ educational data	Degree	1	4%
	Diploma	3	12%
	Grade 11–12	13	50%
	Grade 8–10	5	19%
	Grade 4–7	4	15%
Gender of children with ID	Males	19	73%
	Females	7	27%
Health problems of children with ID	Epilepsy	4	14%
	Speech	10	37%
	Physical impairment	2	8%
	Hearing	3	11%
	Dental	7	30%

Most participants were females, 85%, showing that caring for children with ID was on their shoulders. Most men migrated to cities looking for jobs. Half of the participants left schooling before Grade 12, while few obtained tertiary qualifications. Participants indicated a need for more funds as a contributory factor for not proceeding to tertiary education. Most participants were unemployed at 61%. The interview guides helped the researcher to profile information about the children, including age, gender, and other health problems. In addition, the researcher asked participants for more details of medical treatment for those experiencing other health problems besides ID diagnosis. Participants reported their children with ID experiencing comorbidity problems, including speech and hearing, dental and epilepsy, requiring health care support. Female-headed households were at 38%, and single parents at 42%. Most children were males with fewer father figures. The ages of the children were between 6 and 17 years and mostly experienced speech problems at 37% and unattended dental issues, including missing teeth and tooth decay at 30%, requiring health care services. The dental problems were mainly related to the difficulty of the children with ID to identify and report their needs to their caregivers compared to their peers without ID.

### Data collection

The researcher conducted 16 in-depth interviews followed by one focus group discussion of 10 family members raising children with ID in a home environment. The individual in-depth interviews enabled the asking of predetermined questions and focus group discussion triangulation to enhance the richness of data. The participants shared their experiences of caring for their children with ID in a rural environment with the first author. All family caregivers signed written consent to participate before the commencement of data collection. Observational field notes complemented capturing pertinent data not documented through the audio recorder, including the participants’ emotional expressions towards answering questions. Participants gave verbal consent for their narratives to be audio-recorded. The researcher maintained the anonymity and confidentiality of participants throughout the data collection process to protect their human dignity.

Semi-structured interview guides directed the interviews and focus group discussion to explore family caregivers’ experiences raising children with ID in rural communities. The researcher used professional assistance in translating interview guides into the Sepedi language, which is dominant in the district and understood by participants. The researcher was fluent in languages preferred by the participants. The interview guides for in-depth interviews and focus group discussion collected socio-demographic information under section A. The central question was asked about the experiences of raising a child with an intellectual disability, followed by probing questions. The guides enabled comparability amongst data sets of focus group discussions and in-depth individual interviews.

### Data analysis

The researcher analysed data concurrently with the collection process. The inductive thematic data analysis with the assistance of the Atlas Ti software program followed qualitative analysis steps (Rubin & Rubin, 2012) [34] of familiarising oneself with data, generating initial codes, searching emerging themes, reviewing them, and naming their related and subthemes. Data analysis consolidated individual in-depth interviews and focus group discussion data. The first researcher (MJM) translated data into English and transcribed audio-recorded information from both data sets on an Excel Sheet. The researcher read written transcripts with written observational field notes to acquaint oneself with the collected data. The observational field notes maximised analysis in documenting participants’ emotional and psychological status attached to their narratives, further increasing reliability and validity. The researcher allocated various codes to participants according to associated themes. The researcher removed any information linking participants to data to ensure anonymity and confidentiality. Transcripts were loaded to Atlas Ti software to arrange similar codes using the coding manager. The Atlas Ti assisted the researcher in handling multiple overlapping codes without losing their contexts. The researcher conducted content analysis to determine themes and subcategories from frequently occurring styles and patterns in the participants’ narrative statements. Data from both participant groups were duly categorised and compared, including examination of any connections, regularities, variations and peculiarities. The researcher summarised information into meaningful units, presented in thick descriptions and quotes from the participants to demonstrate their authenticated voice in the context of supporting literature-based evidence [35]. The academic supervisor of the study confirmed the appropriateness of the findings.

## Results

The researcher comparably consolidated data analysed from 16 in-depth individual interviews and one focus group discussion of 10 female family caregivers raising children with ID. Table 1 presents the profiles of participants and their children with ID. The results revealed information needs regarding the care of children with ID among family caregivers in rural areas. Most families showed a quest for information regarding the care of children’s challenging behaviour and available resources to enable the caregiving of children with ID. The researcher allocated participants’ narrative numbers, such as Participant 1, age, relationship to the child and the type of interviews, to ensure their anonymity and confidentiality throughout the study. Table 2 presents the generated themes and subcategories of the results.

**Table 2:** Themes and subcategories on understanding challenges

Theme	Subthemes
Caregiving of challenging behaviour of children with ID	Impaired social interaction of the child Impaired emotional well-being of the child Overprotectiveness towards the child Caretaking attachment towards the child
Available child and family support services	Counselling services Social work services Psychological services Health care services for the child

### Caregiving of challenging behaviour of children with ID

This theme emerged during focus group discussion around their experiences of the challenging behaviour of children with ID. In addition, the in-depth individual interview responses on supporting families raising children with ID highlighted that most children display challenging behaviour, and most participants reported difficulties in handling them. Participants communicated the theme as a way to make sure that their children were always safe. The results showed the need for information to understand the care of their children’s challenging behaviour. Family caregivers needed information regarding impaired social interaction of the child, overprotectiveness and caretaking attachment toward their children with ID. The diagnosis of ID limits the intellectual adaptive behaviour of children with ID, including impaired social and emotional well-being that resulted in some family caregivers being overprotective and developing a caretaking attachment to approach and cope with the care of their children.

#### Impaired social interaction of the child

Some participants were concerned about their children’s poor social interaction challenges within and outside the home environments. Most children reported being more comfortable staying at home with familiar family members. The families found introducing these children to new faces, including their peers, difficult.

*“My child doesn’t want to meet people outside the family members. He is more comfortable to stay home” (Participant 5, mother, 63 years, focus group).*

“*My child is having a problem refusing to be sent to the shop near home to buy bread, and he will tell you to send his younger sibling. We cannot force him*”. *(Participant 16, mother, 54 years).*

*“My child doesn’t want to go anywhere. He stays at home. He does not want to visit anywhere, even his sister. I even left him alone at home now” (Participant 12, mother, 43 years, individual interview).*

Some families needed more information to understand that intervening in children’s behaviour can improve with regular medical help provided by professionals. However, some participants found it challenging to associate refusing to be sent or going anywhere outside the home with impaired social interaction, where the child experiences challenges meeting unfamiliar faces. In addition, participants believed that the children decide to stick to the family environment, including disobedience. In other instances, rejection by community members related to stigma can result in poor interaction of the children with ID with other community members outside the home.

#### Impaired emotional well-being of the child

The families highlighted the information to deal with different emotions and mood disorders displayed by their children with ID. Some participants indicated emotional dysregulation among the children with ID, contributing to their stress levels as family caregivers. Speech problems experienced by children with ID resulted in most children not being able to express their needs, thus leading them to display challenging behaviour, such as crying for attention.

*“The child does not want to stay alone, but she needs attention. She will cry even when nothing is happening to her. I mean like crying unexpectedly. I need training on how to treat her properly” (Participant 23, father, 55 years, individual interview)*.

*“I need more information on how to take care of the child. Especially to manage her when she is angry because of her strange behaviour. I will be happy if they can teach me so I don’t stress too much” (Participant 9, aunt, 42 years, focus group).*

Participants believed that adequate information on the care of children with ID would help control and manage their children’s challenging behaviour. These will further reduce the caregiver’s stress level and increase their caregiving skills and self-efficacy towards the difficulties related to the conduct of their children with ID.

#### Overprotectiveness towards the child

Some families displayed and reported an overly protective approach towards their children with ID. Family caregivers gave individual attention to children more than other children at home. The families were concerned that their children with ID need more protection as they cannot stand for themselves. Participants showed the need for information to be able to treat their children equally.

*“I must pay attention and listen to the child more than anyone in the family because he is not able to take care of himself.” (Participant 11, guardian, 27 years, individual interview).*

*“I have other children besides him. I don’t have time for other children; I think I give more attention to him only; I need training to know how to treat other children because this causes conflict”. (Participant 1, mother, 44 years, focus group).*

The delayed development of ID in self-care of the children resulted in dependency of the children on the care and support of family caregivers. However, some families felt that the children should not be treated like other children and care for the children more than their siblings. These could further contribute to the rejection of the child by siblings. Social stigma and lack of inclusiveness of the children with ID could contribute to the family caregivers feeling responsible for protecting their vulnerable children more than any other children. Thus, there is a dire need for information to enable family caregivers to provide care of children with ID without feeling guilty and neglecting the parental care of other children.

#### Caretaking attachment towards the child

Some families reported that children were close to their family members and felt more comfortable around their mothers as caregivers. However, some family members found it difficult to share the caretaking responsibility of the children with other persons willing to help.

*“He refuses to sleep with his siblings. He cries to share a bed with me, and it is not easy because I am married” (Participant 13, mother, 35 years, individual interview).*

*“I do not believe in taking the child to institutions like boarding. I think that the child will think that I don’t like him and have abandoned him” (Participant 16, mother, 54 years, individual interview).*

Family caregivers communicated caretaking attachment to ensure that children receive full support related to their dependency on the family members’ care. Some felt responsible for providing care independently and needed more trust in other facilities with trained professionals to care for and manage their children with ID. Both children and family caregivers found it difficult to wean from caretaking attachment. However, some responses indicate that caring for a child with ID could bring intense bonding, impacting the family’s quality of life. These narratives highlight the need for professional family interventions.

### Available child and family support services

The researcher generated this theme from both focus group discussions and in-depth individual interviews on responses around the available resources to care for and support the families caring for and raising children with ID. The results showed that family caregivers need information on services available for their support as primary caregivers and their children with ID. Support services enable caregivers raising children with ID to cope with the challenges and burdens of care.

#### Counselling services

This study found that most families needed information to access critical health and well-being services involving counselling as professional assistance in public institutions, which is provided free to such families. Some participants reported an inability to cope with the stress of meeting the needs of their children with ID, especially single parents who experienced difficulties meeting their children’s needs. However, they needed the information to access such support services.

One mother who separated from her husband displayed a dire need for counselling services to deal with caring challenges. Her narrative and observational field notes highlighted the frustration of dealing with her child’s care without her partner’s support. The result communicates the need for continuous counselling to enable ongoing support of parenting skills needed to care for the child with ID in a home environment.

*“I was not coping and needed counselling for this matter. But did not know where to go for help.” (Participant 21, mother, 32 years, individual interview).*

Some participants reported difficulties in dealing with the fact that their children were diagnosed with ID. The narratives indicate the information needed for counselling services to enable coping abilities.

*“I just accepted, but at the beginning, I was not coping. I always take whatever is happening and take it very lightly. I still have unanswered questions. It is difficult to let it go” (Participant 10, mother, 45 years, focus group).*

*“I am not sure of the services [counselling] provided to such families; what I know is that we do not have services for the children” (Participant 11, guardian, 27 years, individual interview).*

Some participants believed that information on counselling services would enable them to access professional support for reducing stress levels and using adaptive coping mechanisms to deal with caring responsibilities. Mothers of children with ID mostly communicated the need for counselling services.

#### Social work services

The family caregivers needed access to information to understand the social workers’ services as a support system for their children’s needs. Some used these services for other family problems but less for raising children with ID. They needed information on collaborations between themselves and the social workers to support and provide care to their children with ID.

“*I consulted the social worker in 2012 when we had family issues. The social workers have never visited my child. I even asked them to visit my house, but they never came” (Participant 14, mother, 42 years, individual interview).*

“*We once decided to apply for him to be institutionalised but did not succeed*” (P*articipant* 25, uncle, 31 years, *individual interview).*

Some families were willing to institutionalise their children with ID for different reasons. However, they needed more information on the processes to follow. In most cases, such an institutionalisation process in South Africa is coordinated and finalised through social work services. The communicated evidence further showed the need for home visits by health professionals that will improve individualised family interventions on the information needed for the child’s care. Socio-demographic information revealed that most participants utilised the social work services when making applications for the children’s dependency grants but needed help to continue with the services for information regarding further assistance in the care of such children.

#### Psychological services

Some families were not coping with the diagnosis of their children with ID. The burden experienced by these families leads to some willing to end their lives to deal with the impact of raising a child with ID. One single mother of 5 children was emotional when sharing her experiences:

*“I was frustrated and wanted to ingest poison and kill both my children. I felt the pain” (Participant 14, mother, 42 years, individual interview).*

*“I feel emotional pain that he is like the way he is, not the same as other children” (Participant 18, mother, 50 years, individual interview).*

The excerpts above show evidence of hopelessness related to the burden of raising a child with ID. The care burdens required psychological intervention services to assist the needy family caregivers. The observations on these parents displayed signs of emotional and psychological drains of raising a child with ID. The diagnosis of children with ID showed frustration, especially among mothers who experienced emotional and psychological impacts.

#### Health care services for children with ID

Most children experienced health-related challenges that required intervention in health services such as medical, dental, speech and hearing, and mental health services. Some families needed to utilise community facilities such as clinics to support their children’s basic needs with ID effectively. However, few participants reported taking their children to health care services. Stigmatisation resulted in some family caregivers not being at ease in seeking help to bring their children to health care professionals. However, services for such children are provided freely in South African public institutions.

*“My child shuffles on the floor and cannot walk or sit. I took him [the child] to the church to receive healing. I think mental problems get different treatment from other diseases. I don’t know the services around us to help my child.” (Participant 20, mother, 47 years, individual interview).*

*“I sometimes fail to understand what he wants, especially around people. I get frustrated because I do not understand the sign language that he is using. It is challenging to understand him. (Participant 1, mother, 44 years, focus group).*

*“She lost most of her teeth, and it was difficult to save them. They started by decaying, and most of the time, she refused to chew hard food, complaining of pain. I didn’t take her to the hospital as the tooth fell without anyone removing it. Now, she eats eggs” (Participant 19, mother, 36 years, individual interview).*

*“I wake up during the night when he is fitting. I can hear him struggling when I am asleep. I must make sure that everything is right with him. The child is not on treatment.” (Participant 6, mother, 42 years, focus group).*

The evidence shows that some family caregivers needed information to understand the communication displayed by children with speech problems to respond to their basic needs. Such children require caregivers’ knowledge of primary sign language to facilitate training and rehabilitation of their developmental needs. Lack of adequate information on ID resulted in some families finding alternatives for medical care, such as spiritual and traditional care, for assistance with their children’s health challenges with ID. The dental problems comprised the nutrition of the children as they could not eat hard food, exposing the children to malnutrition.

The results evidenced that although South Africa provides free healthcare services to all citizens at primary healthcare facilities, access to specialised professionals still needs to be improved. Most children experienced health challenges, including epilepsy, physical impairment, speech and hearing problems and dental issues requiring special attention. The study indicates that disseminating information on available ID resources will empower families to provide needed care to their children.

## Discussions

The study explored the information needs of family caregivers regarding the care of children with ID in rural home environments. The results established that most families as primary caregivers experienced information challenges regarding caring for their children’s challenging behaviour and the available support resources in their communities. Furthermore, the need for more information on social support structures in rural areas environment regarding the care and raising of children with ID restrains the inclusiveness of these children to participate in societal activities [36].

Family caregivers needed information to understand and handle challenging behaviour and grooming of their children to possible independence. In support, past research found that parents seek information on the care of their children with ID from professionals to cope with the burden [37]. On the other hand, research revealed that the knowledge of families of children on ID mostly depends on the type of information provided by the professionals educating them [38]. In line with the findings, the previous research found a lack of dissemination to family caregivers of children with disabilities in welfare information by the Indian government [39]. The results extend to prior studies that found information increasing, knowledge and understanding of the parents’ level of children with ID, further leading to self-advocacy in proactive decision-making on the care [40].

Previous research that resonates with this study found that knowledge is good if shared with caregivers who require it to meet the children’s developmental needs [41]. However, the empowerment of families of children with ID is mainly determined by their economic status and educational level [42, 43]. In support, previous results indicate that the caregivers’ awareness of ID is allied to resilience ability, which mainly enhances the care of children with ID [44].

This study is congruent with the reflection that reported that caregivers found the behavioural needs of their children with ID challenging to fit into other environments and required expertise to handle them [45]. Behavioural challenges in ID may indicate the children need attention regarding stimulation, pain reduction, social escape and tangible reinforcement [46]. Some children presented with impaired social interaction with other family and community members. Similarly, it was revealed that children with ID have few interactions with other people and experience rejection or isolation [47]. This finding supports the study established that the environmental and social enablers, including inclusivity in activities by their communities, can determine the children’s social interaction [48]. Congruent with these findings, previous results found that information given to the family caregivers on the children’s behaviour, including emotional changes and aggression, improved their skills to handle the child and further increased feelings of competence and confidence [49].

Most family caregivers resorted to coping mechanisms that involve caretaking attachment and overprotective towards their children with ID. This finding resonates with the research that found that parents with less information on ID were inadequately involved in modelling behaviour that was adaptive to the development of their children with ID [50]. The study results support the finding that Australian children with ID experience emotional and behavioural problems challenging to their caregivers [51]. In addition, the conclusions aligned with the study, which found that in Spain, parents felt responsible for the happiness of their children and developed an overprotective approach towards their care [52]. Comparably, a caretaking attachment bond mainly develops between the child and caregiver based on love, interaction, and the child’s feeling more uncomfortable in the caregiver’s absence [53]. Hence, the overprotectiveness of children with ID sometimes makes it difficult for caregivers to combine their work life and the care of their children with ID [52]. However, a similar study revealed that households with extended family members share the care responsibilities for children with ID, reducing the caring burden [54].

The study highlighted the need for information to empower the family caregivers on the services available in the communities to offer them support, including counselling, social work, psychological care, and health care services for their children. The findings further resonate with the study, which reported that the caregivers of children with ID needed to be made aware of the support resources available to their health needs [25]. Most families with children with ID are highly vulnerable and require support interventions for their well-being [39]. This study highlights that family caregivers, primarily mothers, need information on counselling services to cope with the care challenges of raising their children with ID. In support of this finding, a study conducted in South Africa indicates that most children with developmental disorders were under the care of mothers who are primary caregivers [55]. Similar research established that counselling of families of children with ID decreases their stress levels, increases their self-esteem and further reduces the risk of disorders related to anxiety [56]. Furthermore, congruent with these findings, a study revealed that counselling was crucial in enabling family members to create a friendly, positive home environment for their children with ID [57].

The information needed for health care services included medical, dental, and speech challenges experienced by the children with ID. Some family caregivers reported their children presenting with dental problems such as loose and missing teeth. Comparable studies found that caregivers could identify the dental pain of their children with ID through salivation and putting hands in their mouths more often than their typical peers [58]. In addition, congruent with this finding, the speech of children with ID is more emotional and requires stimulation than their typical development peers [59]. In alignment with this finding, accessing appropriate professional services in rural Australia was challenging due to geographical distances [60]. In support, the study in South Africa revealed that public facilities experience a long list of bookings [61, 62]. These reduce access to available rural support services and resources to cater for the needs of the children with ID and their families.

### Suggestions and recommendations for practice

The study recommends prioritising the information needs of children with ID and their families to receive professional assistance. Policymakers should establish, support, and provide resources on awareness of ID within health and education facilities. Furthermore, the development of directive policies and regulations should strengthen the implementation of education and training programmes on ID awareness within societies. Collaboration of health care professionals and essential service providers, including teachers and educators in early childhood development centres and schools, should support the dissemination of information on ID to the public. In addition, the results recommend ID awareness campaigns and community education regarding the care of children with ID and the support of caregivers. Thus, training nurses, midwives, social workers, and other health professionals, including educators, is vital to enable them to provide ID awareness and train family caregivers. Training of available community healthcare workers and traditional and religious leaders on ID will equip them with the knowledge to be able to support the vulnerable family caregivers of children with ID. These will provide the families with continuous, valuable, updated information to help their children.

Furthermore, the study highly recommends collaborative professionals for training, empowerment, and providing psychoeducational interventions that will strengthen caregiver competence and confidence in ensuring continuity of the caregiving of children with ID from the home environments. Collaboration and coordination of educational facilities, social development, rehabilitation, and health care services will further enhance information on ID for the public. The study suggests that professionals should actively facilitate forming community support groups and home-based care services to interact and equip family caregivers with relevant information regarding ID awareness to reduce social stigma. Forming partnerships with family caregivers will strengthen information giving and access.

The study’s limitations involve data on the prevalence of children with ID in Limpopo Province that were not available. The study could not capture the experiences of family caregivers of children with ID not accessed through snowballing and known to community health care facilities. Fathers of the children with ID were less presented, limiting their narrative experiences on the caregiving of their children. The study has no information on professional facilities available for support services in rural communities.

## Conclusion

This study provides insights into the information access and understanding of family caregivers of children with ID in rural areas of Limpopo Province. The study findings indicate the need for information access by these families to understand the care of their children with ID. The study shows the crucial responsibility of service providers to activate the information support system of caregivers raising children with ID in rural home environments. The findings of this research hope to inform the establishment of training programmes to improve ID information by empowering and enabling family caregivers in rural areas to improve understanding of the care of their children with ID. Training programmes that continuously update caregivers’ knowledge of ID play a critical role in enhancing self-efficacy and competency to support their children. The study supports stakeholders’ collaboration on ID awareness campaigns and opens more platforms to disseminate information and educate the public, including those in rural areas with fewer resources.

### Electronic supplementary material

Below is the link to the electronic supplementary material.

Supplementary Material 1

## Data Availability

The datasets used and analysed during the current study are available from the corresponding author upon reasonable request. The data are not publicly available due to information that could compromise the privacy of the research participants. Please write to juwamod@gmail.com.

## Abbreviations

ID: Intellectual disability
