# Supplementary material for: Exploring information needs among family caregivers of children with intellectual disability in a rural area of South Africa: a qualitative study
Source: BMC Public Health. 2024 Apr 24;24:1139. doi: 10.1186/s12889-024-18606-7 (PMC11040777; doi:10.1186/s12889-024-18606-7)
Supplement: Supplementary file 1 — Supplementary Material 1 [file 12889_2024_18606_MOESM1_ESM.pdf]

SECTION A: SOCIO-DEMOGRAPHICS

Kindly provide the following information in terms of Demographics:

Information about the child

1. Age of the child in years…………………
2. Gender of the child □ Male □ Female
3. What other diagnosis has your child received? ………………………………….
4. Grade of the child ………………………………………………………………
5. Type of the school ………………………………………………………...............
6. Grant support ……………………………………………………………………
7. Is this the first child with the diagnosis? □ Yes □ No

Information of participants

8. Age ………………………….
9. Gender ………………………………...
10. Relationship to child: □ Mother □ Father □ Sibling □ Guardian □ Uncle □ Aunt

    □ Grandmother □Grandfather
11. Educational Qualifications …………………………………………………………
12. Religion……………………………………………………………………………..
13. Marital status □ Married □ Divorced □ Separated □ Widowed
14. Employment status ………………………………………………………………
15. Source of income …………………………………………………………………

SECTION B: INDIVIDUAL IN-DEPTH INTERVIEW

INFORMATION REGARDING THE CHALLENGES AND SUPPORT NEEDS

Please provide detailed information regarding the experiences of living with the children with ID and support needs you expect to receive. (Guidelines below)

1. Do you have access to relevant information or trained about care of the child with intellectual disability? **Yes/ No**

1.1 If yes please elaborate
……………………………………………………………………………………………………
……………………………………………………………………………

2. What is your role/contribution do you offer as a family member to care for your child with ID?
……………………………………………………………………………………………………
……………………………………………………………………………………………

3. Did you receive any form of counselling? **Yes/ No**

   3.1. If yes, please elaborate

……
……………………………………………………………………………………

4. How do you as a whole family cope with and manage the effects of living with a child diagnosed with ID?
……………………………………………………………………………………………………
……………………………………………………………………………………………

5. Explain your family's professional relationships ……………………………..................

………………………………………………………………………………………………

6. Are you receiving any support from relatives/ friends/ community? **Yes/ No**

   6.1. Please elaborate on your answer
   ……………………………………………………………………………………………
   ……………………………………………………………………………………………

7. Explain the assistance you receive from the health professionals in this regard.

……
………………………………………………………………………………

8. What services are available to support your family to meet your challenges?
……………………………………………………………………………………………………
……………………………………………………………………………………………

9. What legal rights do the family have about the care and support of the child?

……
…………………………………………………………………………………………

10. In your opinion, what can be done to help and support the family members in managing living with ID children effectively?

………
………………………………………………………………………………………

11. How does the effect of living with a family member diagnosed with ID make you feel?

………
…………………………………………………………………………………

12. What challenges do you experience as a family, resulting from living with ID child?
    ....
    ....

SECTION C: INTERVIEW GUIDE FOR FOCUS GROUP DISCUSSION

"**Describe your experiences regarding raising a child with intellectual disability"**

1. What is your understanding of the causes of intellectual disability of your child?
2. At what age was your child diagnosed with Intellectual disability?
3. Describe the behaviour of your child.
4. How do you stimulate the child at home?
5. Explain the resources available in the community to enable you to cope with the care of the child.

THANK YOU FOR YOUR PARTICIPATION!!!
